# A mobile device reducing airborne particulate can improve air quality

**DOI:** 10.3934/publichealth.2020038

**Published:** 2020-07-02

**Authors:** Gabriele Messina, Giuseppe Spataro, Laura Catarsi, Maria Francesca De Marco, Anna Grasso, Gabriele Cevenini

**Affiliations:** 1 Department of Molecular and Developmental Medicine, University of Siena, via Aldo Moro 2, Siena, Italy; 2 Department of Medical Biotechnologies, University of Siena, via Aldo Moro 2, Siena, Italy; 3 Post Graduate School of Public Health, University of Siena, via Aldo Moro 2, Siena, Italy; 4 Medical Management, “Le Scotte” Teaching Hospital, viale Mario Bracci 16, Siena, Italy

**Keywords:** healthcare associated infections, surgical site infections, ultraviolet disinfection, UV-C, airborne particulate, air quality, operating room

## Abstract

Surgical site infections are the second major cause of hospital acquired infections, accounting for a large part of overall annual medical costs. Airborne particulate is known to be a potential carrier of pathogenic bacteria. We assessed a mobile air particle filter unit for improvement of air quality in an operating room (OR). A new mobile air decontamination and recirculation unit, equipped with a crystalline ultraviolet C (Illuvia® 500 UV) reactor and a HEPA filter, was tested in an OR. Airborne particulate was monitored in four consecutive phases: I) device OFF and OR at rest; II) device OFF and OR in operation; III) device ON and OR in operation; IV) device OFF and OR in operation. We used a particle counter to measure airborne particles of different sizes: ≥0.3, ≥0.5, ≥1, ≥3, ≥5, >10 µm. Activation of the device (phases III) produced a significant reduction (p < 0.05) in airborne particulate of all sizes. Switching the device OFF (phase IV) led to a statistically significant increase (p < 0.05) in the number of particles of most sizes: ≥0.3, ≥0.5, ≥1, ≥3 µm. The device significantly reduced airborne particulate in the OR, improving air quality and possibly lowering the probability of surgical site infections.

## 1. Introduction

Surgical site infections (SSI) are defined by the Centre for Disease Control and Prevention and the European Centre for Disease Prevention and Control as infections occurring within 30 days of an operation or within one year if an implant [1],[2] and are among the leading causes of death in surgical patients [3],[4].

One of the main airborne pathogens responsible of SSI is *Staphylococcus aureus*. It can cause severe infection of the surgical site and is mostly released by the skin flora of operating room staff [4],[5]. It is a major hospital-acquired infection (HAI) in Europe and the United States (U.S.). In U.S. and EU hospitals, overall annual medical costs of HAI have reached $40 billion, of which a large part is due to SSI [6]. The costs are due largely to prolonged hospitalization, diagnostic examinations, treatment and surgical procedures for re-intervention [7]. As indicated by the systematic review of Badia JM et al. (2017), many studies have shown that the financial burden of surgery is higher for patients who develop SSI than patients without complications [8]. The impact on patient health-related quality of life (HRQoL) is another important consideration: it has been demonstrated that SSI have a negative impact on HRQoL due to prolonged hospitalization and increased morbidity [9].

There is abundant evidence that airborne particles are potential carriers of pathogenic bacteria [10]–[12]. Particles contaminating operating room air may be an additional potential cause of SSI [13]. Although the association between airborne particles and microbes is still debated, electronic particle counting can be considered an objective parameter of the efficacy of air filtering and recirculation systems in operating rooms [14]. Operating room air quality is important, and is directly linked to proper room ventilation and air filtration [15], which is in turn relevant for the reduction of airborne particulate [13].

The International Standards Organization (ISO) classification is used to quantify operating room cleanliness in terms of suspended particles. The scale ranges from ISO 1 indicates the cleanest and ISO 9 the dirtiest air [16].

Gormley T et al. (2017) demonstrated that although operating rooms have a higher ventilation rate, air velocity in different areas of the OR varies significantly, so some points of the room could be more susceptible to microbial contamination, suggesting that “through better or different approaches to air management in ORs, cleaner air can be delivered to key points, such as the instrument table and sterile field, at no more or maybe even less cost” [16].

The purpose of this study is to verify whether a mobile unit for air particle filtration can significantly and usefully improve operating room air quality, particularly in terms of airborne particulate, under real operating conditions.

## 2. Materials and methods

### 2.1. Study design and setting

A cross-sectional study was conducted in March 2018 at Siena University Hospital (Italy), in an ISO-7 operating room, with a volume of 90 m^3^, about 15 air changes per hour and a pressure difference (ΔP) of 5.6 Pascal with respect to the adjacent main door.

### 2.2. Mobile unit for filtering air particles

The device, an Illuvia® 500UV produced by Aerobiotix Inc. (Dayton, OH), is a mobile unit with four wheels that is easily moved by a single person. The unit has a disposable carbon filter cartridge which removes large particles and debris from the incoming air. The air is directed into a chamber with a crystalline structure where it is sterilized by confinated ultraviolet C light, and is then returned to the room through a HEPA filter that removes particulate. The device also monitors particle mass, air temperature and humidity.

As suggested by the manufacturer, the device was positioned with the input close to the main door (sideways) and the output directed towards the center of the OR.

### 2.3. The experiment

The experiment was designed to test whether the device affected air quality. Environmental contamination was monitored in the following four consecutive phases: I) device OFF and OR at rest; II) device OFF and OR in operation; III) device ON and OR in operation; IV) device OFF and OR in operation. The operation conducted was bariatric surgery. Ten theatre staff were in the OR throughout phases II to IV. During phases II, III and IV, the OR door was opened 8, 9 and 4 times, respectively. The door is activated by pressing a button. The opening and closing phases last 5 seconds, while the door remains open for 15 seconds, making an automatic opening-closing time of 25 seconds. The measurements in the OR took place over a period of approximately 3 hours.

### 2.4. Monitoring of environmental contamination

**Figure 1.**
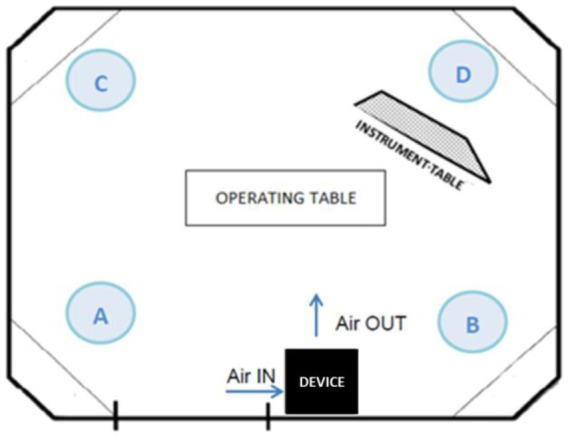
Scheme of the operating room with the position of the air treatment device and the four air sampling points.

To assess the efficacy of the device we used a calibrated Climet Ci-550 particle counter to measure airborne particles of ≥0.3, ≥0.5, ≥1, ≥3, ≥5 and >10 µm. To have a better representation of OR air quality, a time series of measurements were made. Air samples were collected approximately every 5 minutes at four points (A, B, C and D) about 1 meter from the four corners of the OR. Points A and B were on the same side as the device, points C and D on the opposite side (Figure 1).

### 2.5. Database

All the information was entered in a database: sampling time; door opening time; number of theatre staff; Total and stratified airborne particle count for the sizes: ≥0.3, ≥0.5, ≥1, ≥3, ≥5, >10 µm; Phase of the study, and sampling point (A, B, C, D). Microsoft Excel 2010 was used to organize the data and charts.

### 2.6. Statistical analysis

Descriptive and inferential statistics were analyzed with software Stata 12 (StataCorp. 2011. Stata Statistical Software: Release 12. College Station, TX: StataCorp LP). The Wilcoxon rank test for paired data was used to determine whether the device affected the amounts of particles of different sizes, between phases II-III and III-IV. Statistical significance was set at 95% (p < 0.05).

## 3. Results

Table 1 shows particle numbers by size sampled at the four points in the OR in the four phases of the study. All measurements indicated a higher number of particles when the device was OFF. It is also possible to see from the table and the figures that the smallest particles were the most represented and had the highest variations in particulate matter.

**Table 1.** Count of particles of different sizes at four points in the operating room, in the four phases of the experiment with the operating room at rest or in operation.

Point	Particle size (µm)	Operating room conditions and device ON/OFF				
		Rest	Operational			
		Device OFF	Device OFF beginning	Device ON	Device ON	Device OFF end
A	≥0.3	25,354	883,666	49,271	91,992	163,912
	≥0.5	19,048	252,032	36,450	67,703	119,623
	≥1	12,293	78,448	20,251	38,053	60,946
	≥3	1,818	11,403	3,929	7,065	11,009
	≥5	844	5,633	2,262	3,507	5,955
	≥10	100	1,925	880	909	2,197
B	≥0.3	39,056	305,541	56,100	105,357	101,856
	≥0.5	27,001	139,939	40,566	69,507	71,383
	≥1	16,299	56,221	22,484	35,290	37,087
	≥3	2,791	8,611	4,230	5,669	6,535
	≥5	1,202	4,352	2,240	2,877	3,450
	≥10	236	1,639	644	973	1,152
C	≥0.3	13,794	216,347	54,511	55,455	79,815
	≥0.5	9,026	129,623	41,855	41,139	58,992
	≥1	5,562	57,567	25,505	23,465	31,969
	≥3	973	9,148	3,851	4,108	5,032
	≥5	422	4,731	1,732	1,954	2,405
	≥10	100	1,410	257	544	551
D	≥0.3	8,060	119,952	38,733	79,193	84,332
	≥0.5	5,018	84,418	28,125	58,505	62,413
	≥1	2,999	45,348	14,567	32,341	33,007
	≥3	579	8,074	2,412	5,554	5,826
	≥5	257	3,829	1,202	2,734	2,970
	≥10	93	894	422	751	1,037

**Figure 2.**
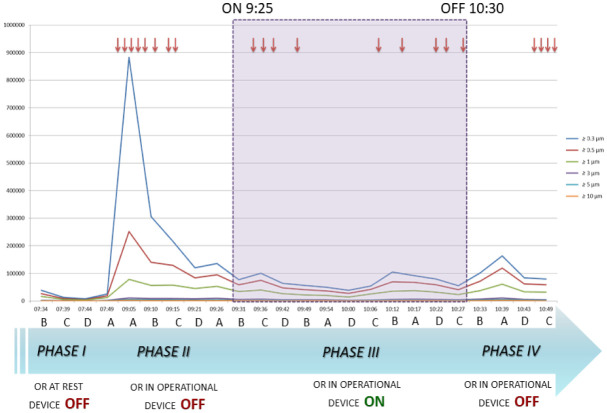
Level of contamination by particle size at the four operating room sampling points (A, B, C, D) and the number of door openings (arrows) in the four phases of the study. The shadowed area indicates when the device was ON.

Figure 2 shows plots of airborne particulate by particle size and number at operating room sampling points (A, B, C, D). The number of particles during phase II was falling and perhapse they continued to decrease. Nevertheless, we noted the followings: i) in the middle of phase III (device ON) particles were more reduced; ii) moving from phase III to IV (device OFF) there was a significant increase (p < 0.05) of particles number. It seems to justify that the device had a role in the control of particle matter.

**Figure 3.**
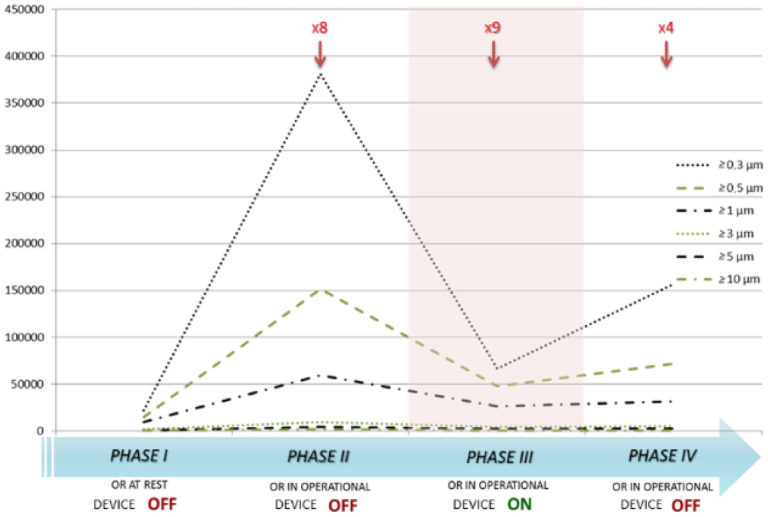
Mean level of contamination by particle size and number of door openings (arrows) in the four phases of the study. The shadowed area indicates when the device was ON.

Figure 3 shows the average number of particles by size for the four phases of the study. There was a statistically significant reduction (p < 0.05) in the number of ≥0.3 µm, ≥0.5 µm, ≥1 µm, ≥3 µm, ≥5 µm, and ≥10 µm particles between phases II (device OFF) and III (device ON) and an increase (p < 0.05) in the number of ≥0.3, ≥0.5, ≥1 and ≥3 µm particles between phases III (device ON) and IV (device OFF).

## 4. Discussion

The study was designed to verify whether the mobile air-treatment device could improve air quality in terms of airborne particulate during a surgical procedure.

At the beginning of the experiment, levels of airborne particulate in the OR were in ISO 7 class, which requires that the concentration of airborne particles ≥0.5 µm at rest be below 352,000/m^3^. When the device was switched OFF and the OR was in operation, particulate matter levels remained below the ISO 7 limit. In contrast, when the device was ON, the air classification level was close to ISO 6. Due to the diluting effect of the treated air, the device demonstrated its effectiveness in reducing most airborne particles, including ≥0.5 µm, commonly used as reference for environmental contamination, despite the presence of ten theatre staff and several door openings. Operators in the OR did not suffer and report any disconfort during the operation due to the device.

Door openings may have an impact on air quality, as the positive pressure of the operating room creates direct flows to the outside when the door is opened. This should avoid contamination of the OR with external air. Positive pressure can in some conditions be cancelled or reversed by opening the door, which leads to failure of OR isolation conditions and increase in airborne bacteria and particles [4],[16].

The points near the door seem to show higher levels of particles, as shown in Table 1 and Figure 2. This is probably due to the opening and closing of the door. The intention of monitoring, randomly, several points of OR was to provide a more comphehensive representation of the particulate matter.

Although the effect of door openings on the increase in particulate matter in each phase was not quantifiable, the similar number of door openings when the device was switched OFF and the OR in operation (8 times, phase II) and when the device was switched ON and OR in operation (9 times, phase III) should have had a similar impact on the experiment when comparing the two phases. Instead, there was a significant reduction in particulate matter that was presumably due to the device. In line with this, when the device was switched OFF while the OR in operation (phase IV), the door was opened 4 times and particulate matter increased (Figures 2 and 3).

Previous studies showed the effectiveness of the device in reducing and keeping down particle count [18],[19], but particle sizes were not differentiated. Our study analyzed variations in particle size and particle sampling in several points in the OR. The active role of the device was highlighted by switching it ON and OFF, as the number of particles increased again in most size classes, except ≥5 and ≥10 µm, when the device was OFF.

The incidence of SSI is 2–5% in patients undergoing inpatient surgery, with an annual incidence in the U.S. of 160,000 to 300,000 cases [20]–[22]. These infections are a major economic burden (prolonged hospitalization, investigation, treatment and operating costs) and affect patients' quality of life. While treating surgical site infections is necessary, a preferable solution is to prevent them.

These infections do not always respond to antibiotics. Antimicrobial resistance (AMR) complicates the treatment and eradication of pathogens. In 2017, AMR infections accounted for 17% of bacterial infections in OECD countries and projections indicate that antimicrobial resistance will increase. Different measures, such as hand hygiene, stewardship programs, rapid diagnostic tests, delay in prescribing antibiotics and media campaigns can have a positive impact on antimicrobial resistance, bring significant improvements in the health of the population and save money because they are cheaper than treatment of the consequences. Improvement in environmental hygiene can be equally effective and cost saving [23]. So additional measures, such as the present device for air purification in operating theatres, are welcome.

Measures to increase OR ventilation do not ensure a reduction in the number of particles and the bacterial load in the room, although they do increase costs [11]. Given all these aspects, the present device could help reduce the probability of SSI by reducing airborne particulate that may be a vector of pathogenic bacteria [9].

Previous studies showed that the UV-C air disinfection and recirculation unit led to a significant reduction in both total particle counts and viable particle counts and a non-significant reduction in CFUs [18],[19].

Further studies are needed to clarify the logical but still debated association between airborne particles and microbes. In this regard, the correlation between the use of the device,the microbiological contamination of the air and surgical site infections should be furtherly investigated.

The study has some limits. Although we monitored four sites in the OR, particle variations on the operating table could be different. It was not possible to measure contamination matter on the operating bed during a real surgery because we would have interfreed with the operation and compromise safety; we partly investigated this aspect with preliminary measurements conducted in the empty OR and under simulated operating condition and still detected a reduction when the device was ON. A real operation with theatre staff would of course have produced different results. Repetition of the study in different ORs and with different surgical procedures would enable better characterization of the device's effects. However, like previous studies [18],[19], the present study showed a substantial reduction in the number of particles. Finally, the number of theatre staff, the air change cycle and pressure differences with respect to outside are other risk factors that could have a confounding effect on the final results. Ten the staff in the OR is crowded conditions. In the study, air changes and pressure differences complied with the standard, whereas in a higher standard OR, the effect of the device may not be evident. The device is presumably more effective under standard/critical conditions. The mobility of the device allows it to be positioned so as to optimize its relationship with the logistic disposition of the theatre staff and the working environment. This is an advantage but must be considered carefully, as wrong positions could even increase the probability of contamination of surgical wounds. Indeed, although ORs have laminar airflow, complex and not easily foreseeable aerodynamic interactions between the structures, the theatre staff and the room itself can induce turbulence and unwanted airflows and introduce an element of uncertainty and difficulty in the correct positioning of the device. This is therefore a critical aspect worthy of careful study in different real scenarios.

## 5. Conclusions

The mobile device for operating room air purification proved effective in significantly reducing the concentration of airborne particulate during surgery, hopefully reducing the likelihood of SSI. Since its effectiveness is likely to depend on where it is placed in the OR, further studies are needed to determine the optimal location in the operating room.
